# Restoration of Decreased T Helper 1 and CD8+ T Cell Subsets Is Associated With Regression of Lymphoproliferative Disorders Developed During Methotrexate Treatment

**DOI:** 10.3389/fimmu.2018.00621

**Published:** 2018-04-04

**Authors:** Shuntaro Saito, Katsuya Suzuki, Keiko Yoshimoto, Yuko Kaneko, Kunihiro Yamaoka, Takayuki Shimizu, Takehiko Mori, Shinichiro Okamoto, Kaori Kameyama, Koichi Amano, Jun-ichi Tamaru, Michihide Tokuhira, Tsutomu Takeuchi

**Affiliations:** ^1^Division of Rheumatology, Department of Internal Medicine, Keio University School of Medicine, Tokyo, Japan; ^2^Division of Rheumatology and Clinical Immunology, Saitama Medical Center, Saitama Medical University, Saitama, Japan; ^3^Division of Haematology, Department of Internal Medicine, Keio University School of Medicine, Tokyo, Japan; ^4^Department of Pathology, Keio University School of Medicine, Tokyo, Japan; ^5^Department of Pathology, Saitama Medical Center, Saitama Medical University, Saitama, Japan; ^6^Division of Haematology, Saitama Medical Center, Saitama Medical University, Saitama, Japan

**Keywords:** lymphoproliferative disorder, malignant lymphoma, regression, methotrexate, T cell subset

## Abstract

**Background:**

Lymphoproliferative disorder (LPD), including malignant lymphoma, is a relatively rare but life-threatening complication in RA patients under methotrexate (MTX) therapy. Spontaneous regression of LPD after MTX withdrawal is regarded as a distinct characteristic in part of such LPDs.

**Objective:**

The present study aimed to investigate the immunological difference in regressive LPD and persistent LPD.

**Methods:**

We studied RA patients who developed LPD during MTX administration (*n* = 35) and clinically matched controls (*n* = 35). The time of MTX cessation was defined as week 0, and LPD patients were divided into two groups according to LPD status at week 12: regressive group (*n* = 22) and persistent group (*n* = 13). Flow cytometric analysis of whole blood samples and serum cytokine assays were conducted for LPD (*n* = 10) and control patients (*n* = 10) at weeks 0, 4, and 12.

**Results:**

There was a significant decrease in peripheral lymphocytes and the proportion of T helper 1 cells (Th1 cells), effector memory CD8+ T cells (EMCD8+ T) and Epstein–Barr virus (EBV)-specific CD8+ T cells at the time of LPD diagnosis, and a significant increase after MTX cessation was observed in the regressive group but not in the persistent group. The expansion of Th1 cells and EMCD8+ T cells significantly correlated with an increase in serum interferon (IFN)-γ concentration.

**Conclusion:**

Changes in Th1 cells, EMCD8+ T cells and EBV-specific CD8+ T cells, which coincided with an increase in IFN-γ, were significantly different between regressive LPD and persistent LPD after MTX cessation.

## Introduction

Methotrexate (MTX) is an anti-rheumatic drug and the gold standard for treatment of rheumatoid arthritis (RA) worldwide (1, 2). However, MTX-associated side effects and certain adverse events can lead patients to abandon the treatment (1, 2). Among these, lymphoproliferative disorder (LPD), including malignant lymphoma, is a relatively rare but life-threatening complication (3, 4). Several studies have reported a high likelihood of developing LPD in MTX-treated patients (5, 6). On the other hand, previous reports revealed that chronic inflammation induced by RA itself is also a risk factor for LPD (7). It is difficult to distinguish between LPDs that develop associated with immunosuppression during MTX therapy and those induced by chronic inflammation or as an incidental complication; however, spontaneous regression of LPD following MTX cessation occurs in 30–70% of cases (3, 4, 7, 8). This had led to the suggestion that MTX may have the potential to cause lympho-proliferation. Our group and others previously reported the link between decreased lymphocyte counts at LPD diagnosis and subsequent restoration after MTX cessation and regression of LPD (8, 9), and suggested the association between activation of immune system and LPD regression.

Here, we investigated changes in lymphocyte subsets and serological factors during LPD regression, and assessed the difference in immune status between patients with regressive LPD and those whose LPD did not regress to investigate the pathogenesis of such LPDs.

## Materials and Methods

### Patients and Data Collection

First, we retrospectively reviewed the medical records of RA patients in our institution from January 1995 to December 2013 and found 25 patients with pathologically defined LPD that developed during MTX treatment. We randomly selected 25 control RA patients without LPD who were treated with MTX in our institution matched for age, sex, MTX dose, and RA duration (LPD:control = 1:1). Second, we prospectively registered RA patients who were diagnosed with LPD while under MTX treatment (*n* = 10) from January 2014 to October 2015; clinically matched controls (*n* = 10) were randomly selected from RA patients without LPD who were administered MTX in the same period. We selected clinically matched patients based on categories of age, sex, MTX dose, and RA duration, and found several candidates for control per cases in our background cohort. And then we unintentionally selected one control RA patient based on patient linked-randomized number. The patients’ blood samples were analyzed in the prospective cohort. In total, data from 35 LPD and 35 control RA patients were analyzed.

This study was approved by the ethics committee of Keio University School of Medicine (approval number: 20110136, 20130246, and 20130364) and the ethics committee of Saitama Medical Center, Saitama Medical University (approval number: 759). In accordance with the Declaration of Helsinki, written informed consent was obtained from the patients who had their blood samples analyzed, but consent from patients in the retrospective analysis of clinical features was waived in accordance with the regulations in Japan.

### LPD Assessment

According to the WHO classification of tumors of hematopoietic and lymphoid tissues (4, 10), LPDs were classified into classical Hodgkin’s lymphoma (*n* = 8), diffuse large B cell lymphoma (DLBCL) (*n* = 18), follicular lymphoma (FL) (*n* = 2), lymphomatoid granulomatosis (LYG) (*n* = 2), mucosa-associated lymphoid tissue (MALT) lymphoma (*n* = 1), NK/T cell lymphoma (*n* = 1), reactive hyperplasia (*n* = 1), and LPD with atypical cell proliferation (*n* = 2). The report of “LPD with atypical cell proliferation (*n* = 2)” was derived from other faculty and had no sufficient information of immunohistochemistry, and could not obtain additional pathological information. The presence of the Epstein–Barr virus (EBV) genome was assessed by *in situ* hybridization for EBV-encoded small RNAs (EBER), and clinical stage was determined using the Ann Arbor staging classification with Cotswolds modifications (11).

**Figure 1:**
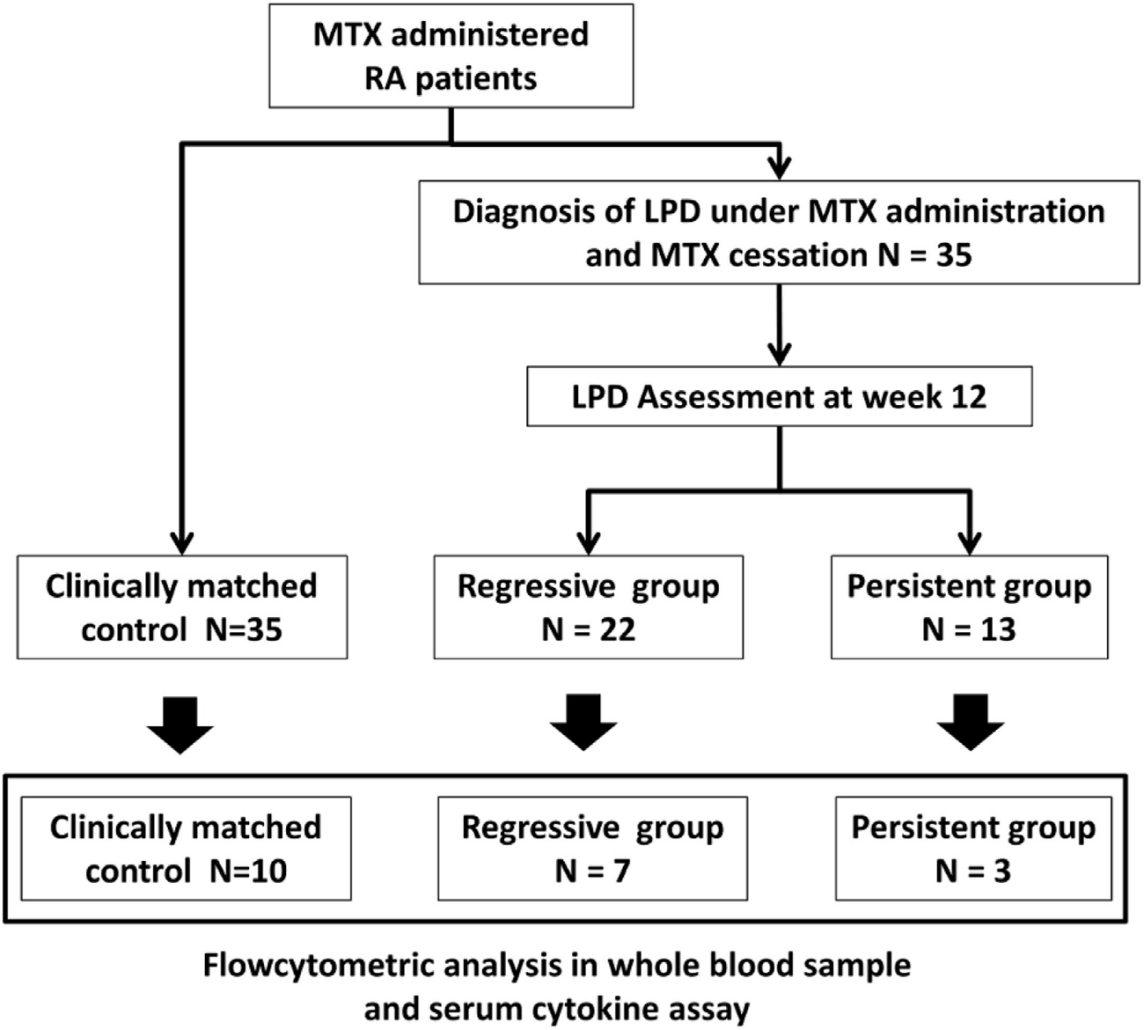
Classification of LPD patients and patient flow. Twenty-two and 13 patients were divided into the regressive and persistent groups, respectively, according to LPD regression assessment at week 12. Control patients were age-, sex-, rheumatoid arthritis duration-, and MTX dose-matched with LPD patients (*N* = 35). Flow cytometric analysis of whole blood sample and cytokine analysis of serum sample were conducted for 10 patients from the control group, 7 patients from the regressive group, and 3 patients from the persistent group. MTX, methotrexate; LPD, lymphoproliferative disorder.

All patients stopped MTX treatment when LPD was suspected. The time of MTX cessation, which was simultaneous with diagnosis of LPD was defined as week 0, and the regression of LPD was assessed at week 12, in accordance with the revised response criteria of the International Working Group (12). Following the assessment of regression, patients were classified into two groups: the regressive group (*n* = 22), those who achieved complete (*n* = 6) or partial remission (*n* = 16), and the persistent group (*n* = 13), those with stable (*n* = 5) or progressive LPD (*n* = 8) (Figure 1). Complete remission was defined as the absence of all evidence of the disease; and partial remission as a LPD regression defined by ≥50% decrease in the sum of the product of the diameters of dominant masses, and no increase in size of other nodes and no new sites (12). Progressive disease was defined as any new lesion or increase by ≥50% of baseline, or cases that received chemotherapy (*n* = 4) or died of LPD (*n* = 1); and stable disease was defined as failure to attain complete or partial response that did not satisfy the definition of progressive disease (12).

We used the last observation carried forward method for the following conditions: intensification of RA treatment, initiation of chemotherapy, and patient death within 12 weeks post-MTX withdrawal. For matched control patients, we analyzed clinical data from the latest visit.

### Flow Cytometric Analysis

Cell surface staining and flow cytometry analysis of fresh whole blood cells were performed at baseline (week 0), week 4, and week 12 for 10 LPD patients and 10 matched control RA patients. Peripheral blood cell subsets were defined by cell surface markers using a standardized method (13). Whole blood cells were stained for 30 min at room temperature in the dark with the following fluorophore-labeled mAbs: anti-CD3-Pacific Blue/fluorescein isothiocyanate (FITC)/PerCP-Cy5.5, anti-CD4-VioGreen (Miltenyi Biotec, Bergisch Gladbach, Germany), anti-CD8- phycoerythrin (PE)-Cy5/PE-Cy7, anti-CD20 allophycocyanin-cyanine 7 (APC-Cy7), anti-CD25-PE-Cy5, anti-CD45RA-FITC, anti-CD56-PE-Cy7/APC, anti-CD127-FITC, anti-chemokine (C-X-C motif) receptor 3 (CXCR3)-PE, anti-chemokine (C-C motif) receptor 6 (CCR6)-PerCP-Cy5.5, anti-CCR7-PerCP-Cy5.5, HLA-A*2402 -restricted EBV-Tetramer-PE, anti-HLA-DR-APC/APC-Cy7 (all from BD Biosciences, Franklin Lakes, NJ, USA), and anti-mouse immunoglobulin G isotype-matched controls (VioGreen from Miltenyi Biotec, the others from BD Biosciences). Stained cells were washed twice with phosphate buffered saline and analyzed on a MACSQuant analyzer (Miltenyi Biotec). The lymphocyte subsets analyzed were CD4+ and CD8+ T cells (including naïve; CD45RA+CCR7+, effector; CD45RA+CCR7−, central memory; CD45RA−CCR7+, effector memory cells; CD45RA+CCR7−). CD4 T cells were classified into Th1 cells (CXCR3+CCR6−), Th2 cells (CXCR3−CCR6−), Th17 cells (CXCR3−CCR6+), and Treg cells (CD25+CD127low). Since detection of EBV-specific CD8+ T cells by the EBV-tetramer was restricted to HLA-A*2402 (14), HLA-A typing was also performed in LPD patients and control RA patients. Data on EBV-specific CD8+ T cells in patients who did not have HLA-A*2402 were excluded from the sub-analysis. Other cell subsets were defined as follows: B cells (CD3−CD20+) and natural killer cells (CD3−CD56+).

### Serum Cytokine Assay and Quantification of EBV Viral Load

The concentration of serum interferon (IFN)-γ and interleukin (IL)-2, IL-7, IL-10, IL-12p70, IL-15, and TNF-α were measured by enzyme-linked immunosorbent assay kits (MSD, Gaithersburg, MD, USA), according to the manufacturer’s protocol. Quantitative EBV-PCR level in whole blood sample were measured by a clinical laboratory testing company (BML Inc., Tokyo, Japan).

### Statistical Analysis

Descriptive values are expressed as medians (Q1–Q3) or range. Comparisons of values and percentages of clinical parameters between the three groups were conducted using the Kruskal–Wallis test and Chi-squared test. Comparisons between two groups were conducted using paired or unpaired Wilcoxon test and Fisher’s exact test. Correlations were analyzed by the Spearman’s correlation coefficient. *P*-values less than 0.05 were regarded as statistically significant. All statistical analyses were performed with JMP software 11.2.0 (SAS Institute Inc., Cary, NC, USA).

## Results

### Baseline Characteristics of Regressive LPD, Persistent LPD, and Control Groups

**Figure 2:**
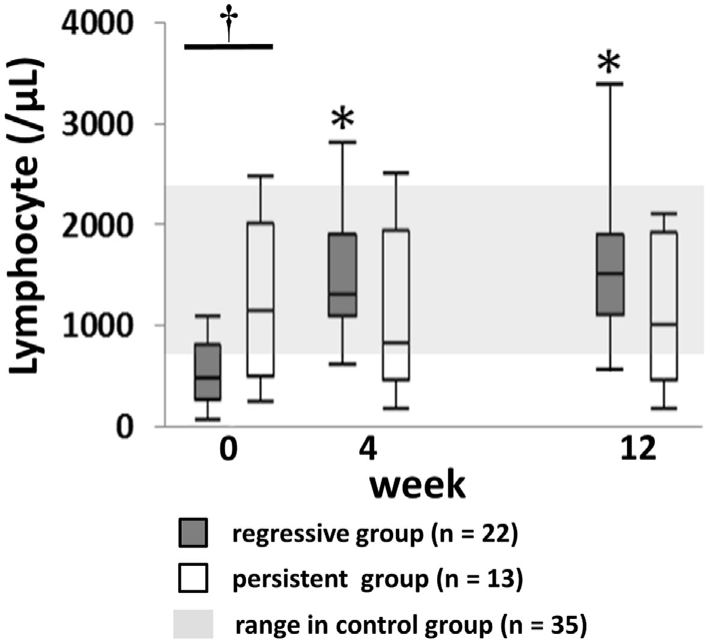
Changes of lymphocyte numbers after MTX cessation in lymphoproliferative disorder (LPD) patients. Change in lymphocyte counts in regressive and persistent LPD compared with the range in the control group. Kruskal–Wallis test was used for comparing the three groups. ^†^Regressive vs. control, *P* < 0.05; regressive vs. persistent, *P* < 0.05. *P < 0.05 for comparison with the value at week 0 in each group. MTX, methotrexate.

The characteristics of patients at the time of LPD diagnosis and grouped into regressive (*n* = 22) and persistent groups (*n* = 13), and the control group (*n* = 35) are shown in Table 1. There were no significant differences in the demographic characteristics and RA features among the groups. Regarding the histological subtype of LPD, the proportion of patients with classical Hodgkin’s lymphoma was lower and DLBCL was higher in the regressive group than the persistent group. The prevalence of EBER-positive LPD was similar between the regressive and persistent groups. Laboratory tests showed significantly higher levels of lactate dehydrogenase (LDH) and C-reactive protein (CRP) in the regressive and progressive groups compared with the control group, but there was no difference between the regressive and progressive groups. Absolute lymphocyte number was significantly lower in the regressive group compared with the progressive and control groups (508, 1,165, and 1,321/μL, respectively, *P* < 0.01). The decreased count of lymphocytes in the regressive group rapidly recovered to a level equivalent with that of the control group at week 4 (1,358/μL) after MTX withdrawal. By contrast, the lymphocyte number in the persistent group did not significantly change after MTX withdrawal (Figure 2).

**Table 1:** Characteristics of LPD and control RA patients.

	Regressive group (*n* = 22)	Persistent group (*n* = 13)	Control group (*n* = 35)	Regressive vs. persistent, *P*	Regressive vs. control, *P*	Persistent vs. control, *P*
**Demographics**						
Age (years)	67 (58–73)	67 (64–71)	66 (57–72)	0.64	0.98	0.66
Female, *n* (%)	20/22 (91%)	11/13 (85%)	31/35 (89%)	0.57	0.78	0.71
**RA features**						
RA disease duration (months)	132 (57–227)	132 (115–298)	155 (88–201)	0.62	0.74	0.95
RF positive, *n* (%)	17/20 (85%)	9/13 (69%)	28/35 (80%)	0.28	0.64	0.43
ACPA positive, *n* (%)	14/18 (78%)	3/7 (43%)	17/23 (74%)	0.10	0.77	0.13
TJC	1 (0–3)	0 (0–2)	0 (0–1)	0.78	0.74	0.48
SJC	1 (0–3)	1 (0–2)	0 (0–2)	0.44	0.47	0.06
MTX duration (months)	65 (23–99)	98 (72–120)	87 (49–115)	0.08	0.17	0.41
MTX dose (mg/week)	10 (6–12)	8 (6–12)	10 (6–12)	0.82	0.61	0.56
MTX cumulative dose (mg)	2,080 (844–3,849)	3,064 (1,810–3,744)	2,760 (1,408–4,144)	0.29	0.46	0.99
Concomitant DMARDs	12/22 (55%)	6/13 (46%)	14/35 (40%)	0.63	0.28	0.70
Sjögren’s syndrome	2/22 (9%)	4/13 (31%)	3/35 (9%)	0.11	0.95	0.07
**LPD features**						
Pathological phenotype (*n*, %) cHL DLBCL FL MALT lymphoma NKT cell lymphoma LYG Reactive hyperplasia LPD with atypical cell proliferation	2 (9%) 14 (64%) 0 (0%) 1 (5%) 1 (5%) 2 (9%) 1 (5%) 1 (5%)	6 (46%) 4 (31%) 2 (15%) 0 (0%) 0 (0%) 0 (0%) 0 (0%) 1 (8%)	– – – – – – – –	0.07	–	–
EBER positive, *n* (%)	13/19 (68%)	7/11 (64%)	–	0.79	–	–
Clinical stage, I/II/III/IV, *n* (%)	10/4/4/4 (45/18/18/18, %)	3/3/5/2 (23/23/38/15, %)	–	0.47	–	–
White blood cell count (× 10^3^/μL)	4.7 (3.0–7.0)	6.1 (4.4–8.3)	5.5 (4.4–6.4)	0.14	0.26	0.52
Lymphocyte count (/μL)	508 (286–825)	1,165 (517–2,035)	1,321 (1,045–1,691)	<0.01*	0.01*	0.55
LDH (IU/L)	226 (186–314)	257 (189–342)	194 (170–226)	0.82	0.02*	0.02*
CRP (mg/dL)	1.48 (0.26–2.88)	0.9 (0.11–2.20)	0.08 (0.03–0.47)	0.31	<0.01*	<0.01*
IgG (mg/dL)	1,218 (1,052–1,451)	1,511 (1,228–1,986)	1,518 (1,195–1,689)	0.05	0.12	0.63
sIL-2R (IU/L)	871 (447–1,436)	1,910 (894–2,600)	–	0.15	–	–

*LPD, lymphoproliferative disorder; RA, rheumatoid arthritis; RF, rheumatoid factor; ACPA, anti-cyclic citrullinated peptides antibody; TJC, tender joint count; SJC, swollen joint count; MTX, methotrexate; DMARD, disease-modifying anti-rheumatic drug; cHL, classical Hodgkin’s lymphoma; DLBCL, diffuse large B cell lymphoma; FL, follicular lymphoma; LYG, lymphomatoid granulomatosis; MALT, mucosa-associated lymphoid tissue; EBER, Epstein–Barr virus-excreted RNA; LDH, lactate dehydrogenase; CRP, C-reactive protein; IgG, immunoglobulin G; sIL-2R, soluble IL-2 receptor*.

*P < 0.05.

The characteristics of the prospectively registered LPD patients (*n* = 7 with regressive LPD, *n* = 3 with persistent LPD) and controls (*n* = 10) are summarized in Table 2.

**Table 2:** Characteristics of LPD and control RA patients for whom flow cytometric analysis of whole blood sample and cytokine analysis of serum sample were conducted.

	Regressive group (*n* = 7)	Persistent group (*n* = 3)	Control group (*n* = 10)	Regressive vs. persistent, *P*	Regressive vs. control, *P*	Persistent vs. control, *P*
**Demographics**						
Age (years)	70 (44–71)	64 (63–66)	68 (59–73)	0.65	0.73	0.40
Female, *n* (%)	6/7 (86%)	2/3 (67%)	8/10 (80%)	0.49	0.76	0.63
**RA features**						
RA duration (months)	78 (20–175)	96 (49–124)	93 (44–137)	0.73	0.63	0.93
RF positive, *n* (%)	3/5 (60%)	2/3 (67%)	6/10 (60%)	0.85	1.00	0.84
ACPA positive, *n* (%)	4/5 (80%)	1/2 (50%)	5/10 (50%)	0.43	0.26	1.00
TJC	0 (0–1)	1 (0–6)	0 (0–1)	0.08	0.30	0.10
SJC	0 (0–1)	1 (0–2)	0 (0–2)	0.33	0.69	0.21
MTX duration (months)	32 (15–78)	96 (8–117)	49 (24–90)	0.43	0.46	0.61
MTX dose (mg/week)	12 (8–12)	12 (8–12)	10 (8–12)	0.88	0.52	0.58
MTX cumulative dose (mg)	988 (558–3,747)	3,712 (304–3,720)	1,700 (876–3,976)	0.80	0.33	0.87
Concomitant DMARDs	3/7 (43%)	3/3 (100%)	5/10 (50%)	0.09	0.77	0.12
Sjögren’s syndrome	1/7 (14%)	1/3 (33%)	1/10 (10%)	0.49	0.79	0.33
**LPD features**						
Pathological phenotype, *n* (%) cHL DLBCL LPD with atypical cell proliferation	1 (14%) 5 (71%) 1 (14%)	2 (67%) 0 (0%) 1 (33%)	–	0.11	–	–
EBER positivity	4/7 (57%)	1/3 (33%)	–	0.49	–	–
Clinical stage, I/II/III/IV, *n* (%)	4/2/0/1 (57/29/0/14, %)	1/2/0/0 (33/67/0/0, %)		0.49	–	–
White blood cell count (× 10^3^/μL)	4.5 (2.7–10.0)	5.2 (4.6–6.1)	5.4 (4.4–9.0)	0.57	0.73	0.87
Lymphocyte count (/μL)	480 (248–648)	2,001 (1,872–2,070)	1,522 (1,077–1,941)	0.01*	<0.01*	0.13
LDH (IU/L)	224 (186–350)	196 (177–238)	223 (176–240)	0.49	0.59	0.61
CRP (mg/dL)	1.19 (0.10–2.10)	0.22 (0.06–0.22)	0.10 (0.04–0.34)	0.14	0.03	0.80
IgG (mg/dL)	1,319 (781–1,532)	1,631(1,511–1,755)	1,191 (858–2,013)	0.10	0.88	0.51
sIL–2R (IU/L)	555 (283–1,210)	289 (206–956)	–	0.48	–	–
EBV-DNA (× 10^3^ copies/mL)	1.0 (0.0–5.0)	0.8 (0.0–0.9)	–	0.36	–	–

*LPD, lymphoproliferative disorder; RA, rheumatoid arthritis; RF, rheumatoid factor; ACPA, anti-cyclic citrullinated peptides antibody; TJC, tender joint count; SJC, swollen joint count; MTX, methotrexate; DMARD, disease-modifying anti-rheumatic drug; cHL, classical Hodgkin’s lymphoma; DLBCL, Diffuse large B cell lymphoma; EBER, Epstein–Barr virus-excreted RNA; LDH, lactate dehydrogenase; CRP, C-reactive protein; IgG, immunoglobulin G; sIL-2R, soluble IL-2 receptor*.

*P < 0.05.

### Lymphocyte Subsets at LPD Diagnosis

The proportion and absolute number of specified lymphocyte subsets at LPD diagnosis in the regressive and persistent LPD groups are summarized in Table 3. We analyzed EBV-specific CD8+ T cells in those with the HLA-A*2402 allele (regressive LPD: *n* = 4, persistent LPD: *n* = 2, control group: *n* = 5). The incidence of HLA-A*2402 was similar to that reported in a previous study in Japan (15).

**Table 3:** Proportion and absolute numbers of lymphocyte subsets.

	Regressive group (*n* = 7)	Persistent group (*n* = 3)	Control group (*n* = 10)	Regressive vs. persistent, *P*	Regressive vs. control, *P*	Persistent vs. control, *P*
**Proportion (%)**						
CD3+ T cells/lymph	68.1 (49.2–78.9)	78.2 (63.6–83.7)	62.2 (50.7–72.2)	0.59	0.36	0.08
CD4+/CD3+T cells	63.0 (44.6–82.2)	70.8 (56.0–77.6)	71.8 (55.3–80.8)	1.00	0.73	0.93
Naïve/CD4+ T	26.6 (24.0–45.7)	31.4 (28.5–36.2)	34.5 (19.1–38.2)	0.65	0.96	1.00
Central memory/CD4+ T	41.6 (29.3–46.1)	36.6 (33.9–44.0)	43.1 (37.4–49.1)	0.66	0.31	0.27
Effector/CD4+ T	1.1 (0.3–4.3)	0.4 (0.3–0.6)	1.4 (0.3–2.6)	0.27	0.81	0.49
Effector memory/CD4+ T	14.2 (6.1–31.6)	18.0 (13.7–25.6)	16.2 (11.9–20.9)	0.65	0.81	0.67
Th1/CD4+ T	14.8 (6.7–17.3)	19.8 (12.4–21.1)	18.1 (17.0–28.9)	0.17	0.02*	0.58
Th2/CD4+ T	65.2 (60.2–72.5)	55.8 (37.5–63.9)	64.0 (52.4–68.7)	0.17	0.46	0.36
Th17/CD4+ T	16.2 (11.7–18.8)	12.9 (8.0–15.8)	11.7 (7.6–13.5)	0.26	0.05	0.71
Th1/Th17/CD4+ T	4.1 (2.6–8.0)	8.4 (7.3–37.2)	6.2 (4.3–11.6)	0.07	0.34	0.14
Treg/CD4+ T	9.7 (3.6–11.5)	6.5 (5.8–7.5)	7.4 (5.6–9.2)	0.17	0.09	0.45
CD8+/CD3+ T cells	28.4 (14.0–49.2)	16.2 (12.9–25.3)	23.3 (14.9–30.7)	0.45	0.66	0.36
Naïve/CD8+ T	26.1 (14.9–40.4)	12.2 (5.1–31.2)	11.2 (9.3–16.5)	0.80	0.01*	0.26
Central memory/CD8+ T	18.3 (10.9–23.9)	16.7 (9.2–20.4)	10.9 (3.6–18.5)	0.82	0.05	0.27
Effector/CD8+ T	19.8 (6.7–30.4)	15.0 (8.2–30.6)	19.1 (11.8–27.5)	1.00	0.73	0.67
Effector memory/CD8+ T	22.5 (15.7–25.4)	34.3 (26.6–40.9)	45.7 (37.0–49.1)	0.02*	<0.01*	0.08
EBV–specific CD8+/CD8+ T	0.1 (0.1–0.2)	0.4 (0.3–0.6)	0.6 (0.4–1.2)	0.11	0.02*	0.56
B cells/lymph	8.8 (1.6–12.1)	3.3 (3.0–17.3)	11.9 (6.8–15.2)	1.00	0.19	0.55
NK cells/lymph	9.6 (5.8–33.1)	7.1 (5.8–9.55)	16.0 (12.5–23.4)	0.49	0.59	0.06
**Absolute numbers^a^**						
Lymphocytes	480 (248–648)	2,001 (1872–2070)	1,522 (1,077–1,941)	0.02*	<0.01*	0.15
CD3+ T cells	288 (153–534)	1,565 (1,317–1,567)	837 (680–1,037)	0.02*	0.01*	0.05
CD4+ T cells	257 (54–439)	1,021 (876–1,109)	639 (447–693)	0.01*	<0.01*	0.02*
Naïve CD4+ T	82 (11–185)	318 (316–321)	185 (121–235)	0.02*	0.05	0.05
Central memory CD4+ T	107 (24–148)	375 (320–449)	271 (237–313)	0.04*	<0.01*	0.02*
Effector CD4+ T	2 (1–19)	4 (3–6)	8 (2–16)	0.82	0.31	0.45
Effector memory CD4+ T	20 (15–45)	157 (140–284)	92 (61–195)	0.02*	<0.01*	0.08
Th1	26 (8–47)	206 (168–209)	114 (82–220)	0.02*	<0.01*	0.27
Th2	189 (38–320)	552 (510–666)	374 (269–487)	0.02*	0.01*	0.06
Th17	36 (7–84)	156 (83–174)	60 (50–85)	0.07	0.20	0.05
Th1/17	9 (2–26)	88 (73–506)	34 (23–79)	0.02*	0.02*	0.10
Tregs	16 (6–37)	78 (64–79)	36 (25–52)	0.02*	0.04*	0.02*
CD8+ T cells	75 (51–97)	212 (201–395)	192 (118–281)	0.07	0.02	0.55
Naïve CD8+ T	19 (9–83)	25 (20–66)	20 (16–32)	0.36	0.46	0.27
Central memory CD8+ T	11 (9–28)	36 (24–43)	20 (10–26)	0.17	0.66	0.08
Effector CD8+	20 (4–22)	30 (17–122)	27 (18–79)	0.17	0.05	1.00
Effector memory CD8+ T	16 (8–28)	69 (49–162)	82 (49–132)	0.02*	<0.01*	0.93
EBV-specific CD8+ T	0 (0–1)	2 (1–3)	2 (1–7)	0.11	0.02*	0.85
B cells	43 (4–110)	62 (60–358)	143 (98–454)	0.37	0.01*	0.55
NK cells	64 (60–99)	142 (109–198)	272 (147–394)	0.07	0.01*	0.27

*Th, T helper; EBV, Epstein–Barr virus; NK, natural killer*.

*^a^Number per 1 mm^3^ whole blood are indicated as absolute numbers*.

*Values indicate median (Q1–Q3)*.

*P < 0.05.

The proportions of three subsets of cells among CD4+ T cells or CD8+ T cells were significantly lower in the regressive group compared to the control group (T helper 1 cells in CD4+ T cells:Th1 cell/CD4+ T (Th1 cell/CD4+ T), 14.8 vs. 19.8%; effector memory CD8+ T cells within CD8+ T cells:EMCD8+ T cell/CD8+ T (EMCD8+ T cells/CD8+ T), 22.5 vs. 45.7%; EBV-specific CD8+ T cells within CD8+ T cells: EBV-specific CD8+/CD8+T (EBV-specific CD8/CD8+ T), 0.2 vs. 0.6%, *P* < 0.05 for all comparisons of between regressive group vs. control group). The absolute numbers of these three subsets were also significantly lower in the regressive group compared to the persistent and control groups (*P* < 0.05 for all comparisons between regressive vs. persistent, and regressive vs. control). By contrast, the proportion of specific subsets of cells was not significantly different between the persistent and control groups. Therefore, we focused on the transition of the three cell subsets after MTX withdrawal.

### Changes in Th1, EMCD8+ T Cells, and EBV-Specific CD8+ T Cells in Regressive LPD and Persistent LPD

**Figure 3:**
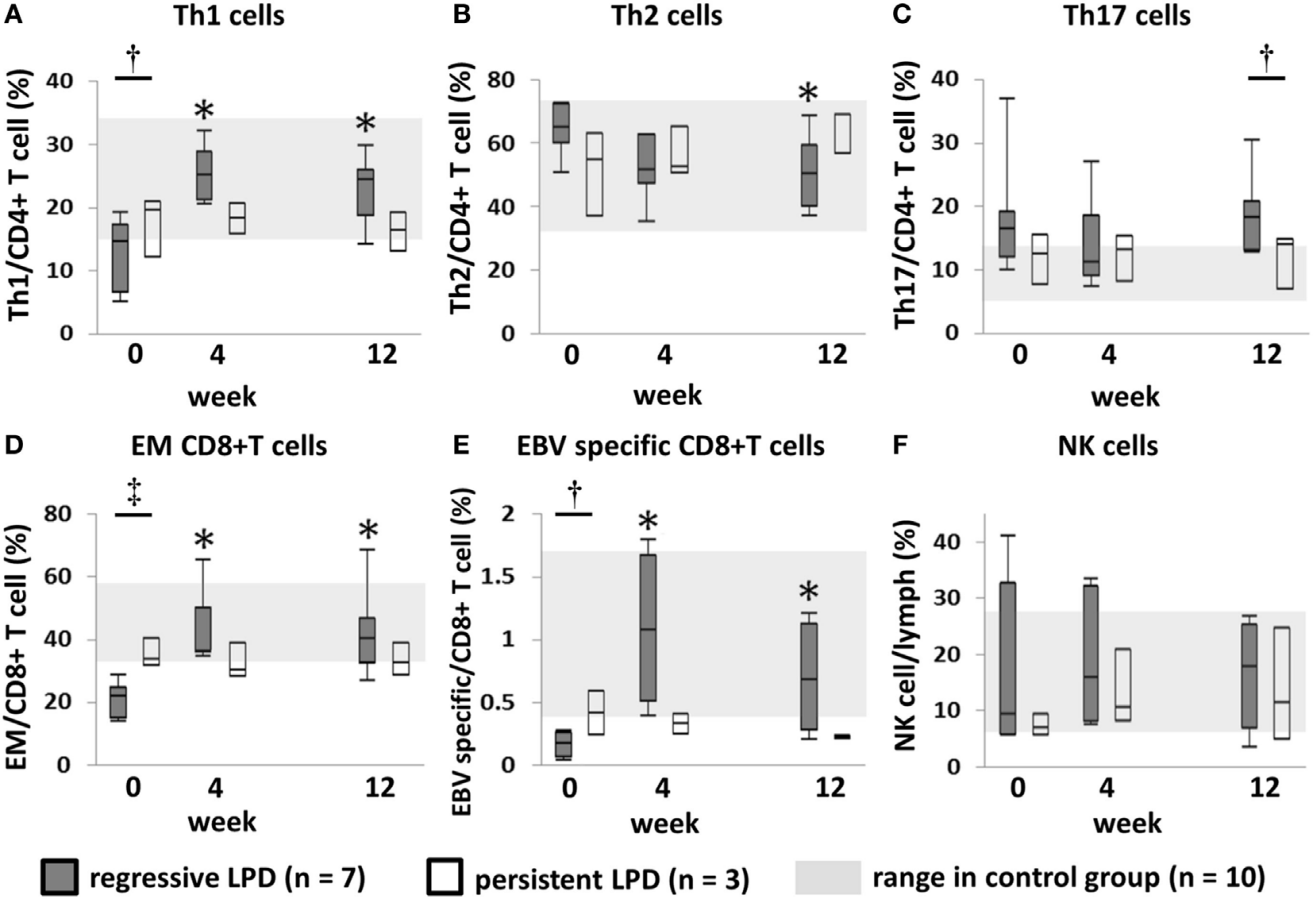
Changes in the proportion of each lymphocyte subset after methotrexate (MTX) cessation. Transition of the proportion of **(A)** Th1, **(B)** Th2, and **(C)** Th17 cells among CD4+ T cells; **(D)** EMCD8+ T cells and **(E)** EBV-specific CD8+ T cells among CD8+ T cells; and **(F)** NK cells among lymphocytes, after MTX cessation. Comparison between the three groups was conducted using the Kruskal–Wallis test. ^†^Regressive vs. control, *P* < 0.05. ^‡^Regressive vs. control, *P* < 0.05; regressive vs. persistent, *P* < 0.05. *P < 0.05 for comparison with the value at week 0 in each group. Th1/2/17, T helper 1/2/17; EM, effector memory; EBV, Epstein–Barr virus; NK, natural killer.

**Figure 4:**
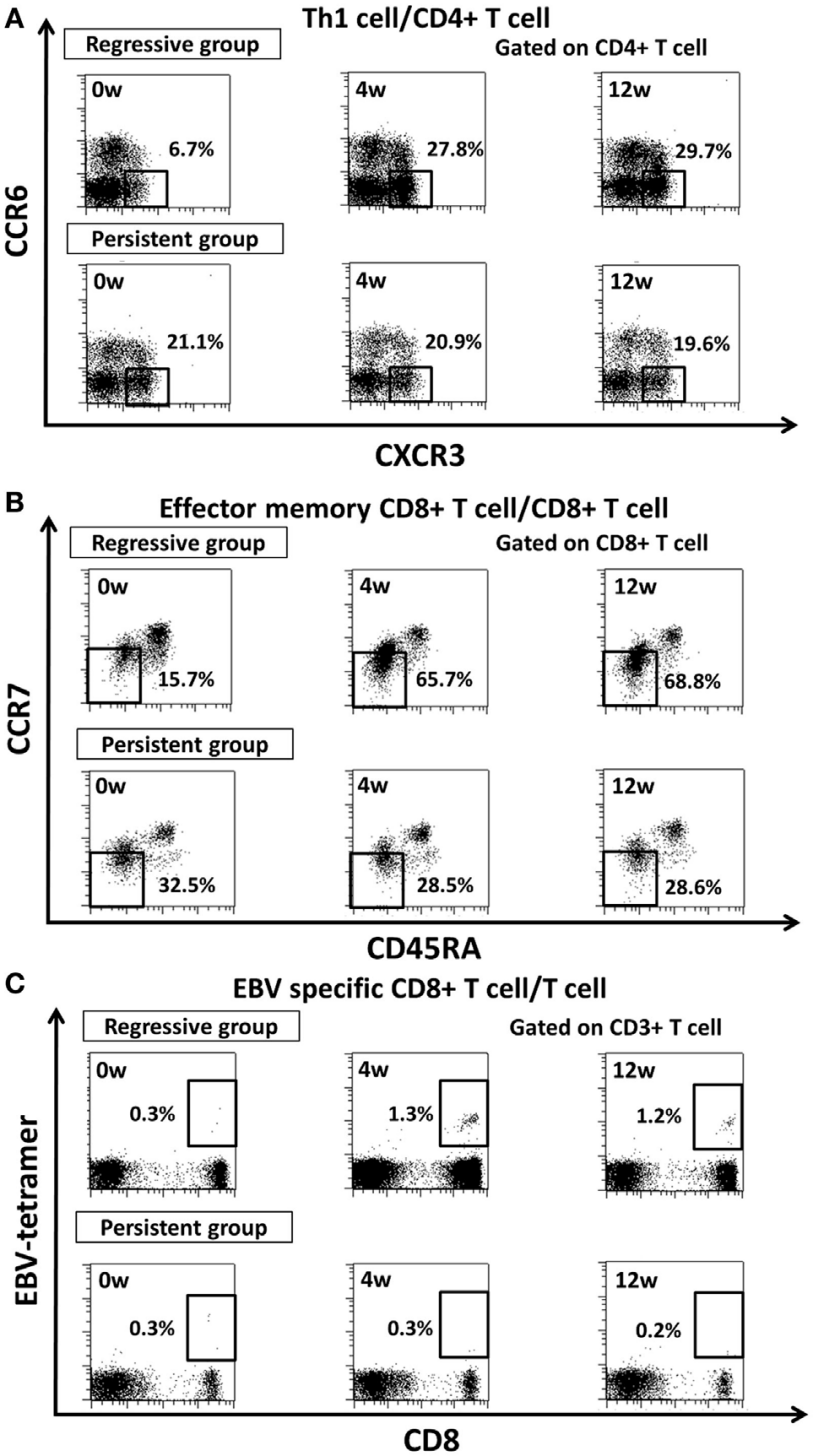
Representative figure for transition of proportion of **(A)** Th1 cell, **(B)** effector memory CD8+ T cell, **(C)** EBV-specific CD8+ T cells after methotrexate cessation in regressive group and persistent group. Th1, T helper 1; EM, effector memory; EBV, Epstein–Barr Virus.

**Figure 5:**
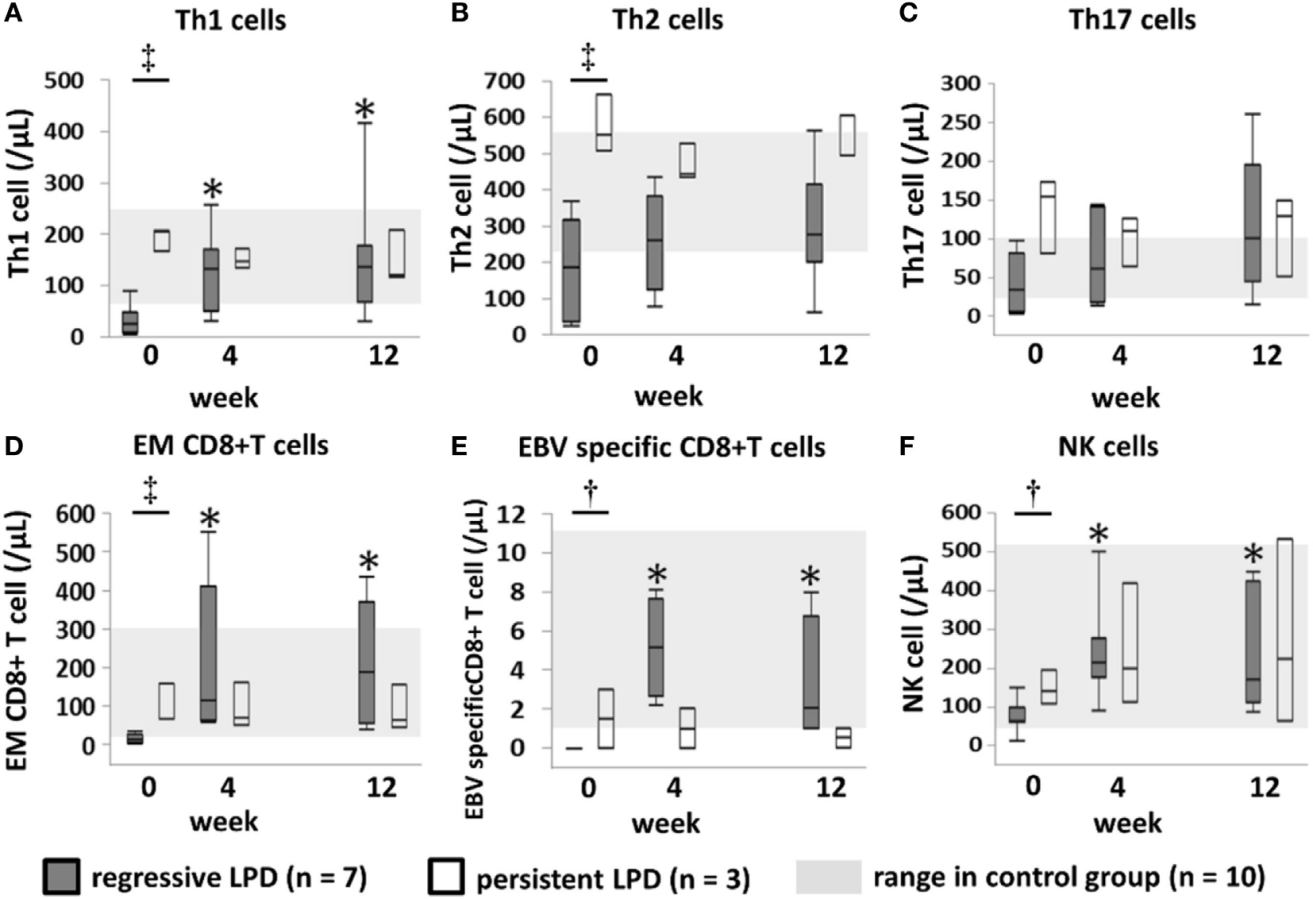
Transition of absolute number of lymphocyte subsets. Transition of the absolute number of **(A)** Th1, **(B)** Th2, and **(C)** Th17 cells among CD4+ T cells; **(D)** EMCD8+ T cells and **(E)** EBV-specific CD8+ T cells among CD8+ T cells; and **(F)** NK cells among lymphocytes, after MTX cessation. Comparison between the three groups was conducted by Kruskal–Wallis test, and comparison between two groups was conducted by Wilcoxon test. ^†^Regressive vs. Control, *P* < 0.05. ^‡^Regressive vs. Control, *P* < 0.05; Regressive vs. Persistent, *P* < 0.05. *P < 0.05 for the comparison with the value at week 0 in each group. Th1/2/17, T helper 1/2/17; EM, effector memory; EBV, Epstein–Barr Virus; NK cell, natural killer cell.

In the regressive group, the proportions of Th1 cells, EMCD8+ T cells and EBV-specific CD8+ T cells increased significantly to reach levels equivalent to those of the control group at week 4, and this was maintained through to week 12 after MTX cessation (Figures 3A,D,E). In the persistent group, the proportion of these subsets of cells was equivalent to the level in the control group at week 0, and did not significantly change after MTX cessation until week 12 (Figures 3A,D,E). The transition of the three subsets is shown in a representative case in Figure 4. In addition, the proportions of Th2 cells, Th17 cells, and NK cells did not significantly change from week 0 to 4 in both the regressive and persistent groups, compared to the control group (Figures 3B,C,F). The changes in the absolute number of these cell subsets are shown in Figure 5. In the regressive group, the absolute numbers of Th1 cells, Th2 cells, EMCD8+ T cells, EBV-specific CD8+ T cells, and NK cells were significantly lower than those of the control group at week 0, but increased to levels that were equivalent with those of the control group at week 4 and were maintained to week 12 after MTX cessation (Figures 5A–F). The absolute numbers of each cell subset in the persistent group were equivalent to the numbers in the control group from week 0 to week 12.

All patients in the regressive group with the HLA-A*2402 allele (*n* = 4) had EBER-positive LPD. Therefore, we could not assess the transition of EBV-specific CD8+ T cells in EBER-negative regressive LPD cases. In addition, the transition of Th1 cells and EMCD8+ T cells was not significantly different between the pathological classifications of LPDs (Figure S1 in Supplementary Material).

### Activation Markers on Effector Memory CD8+ T Cells

**Figure 6:**
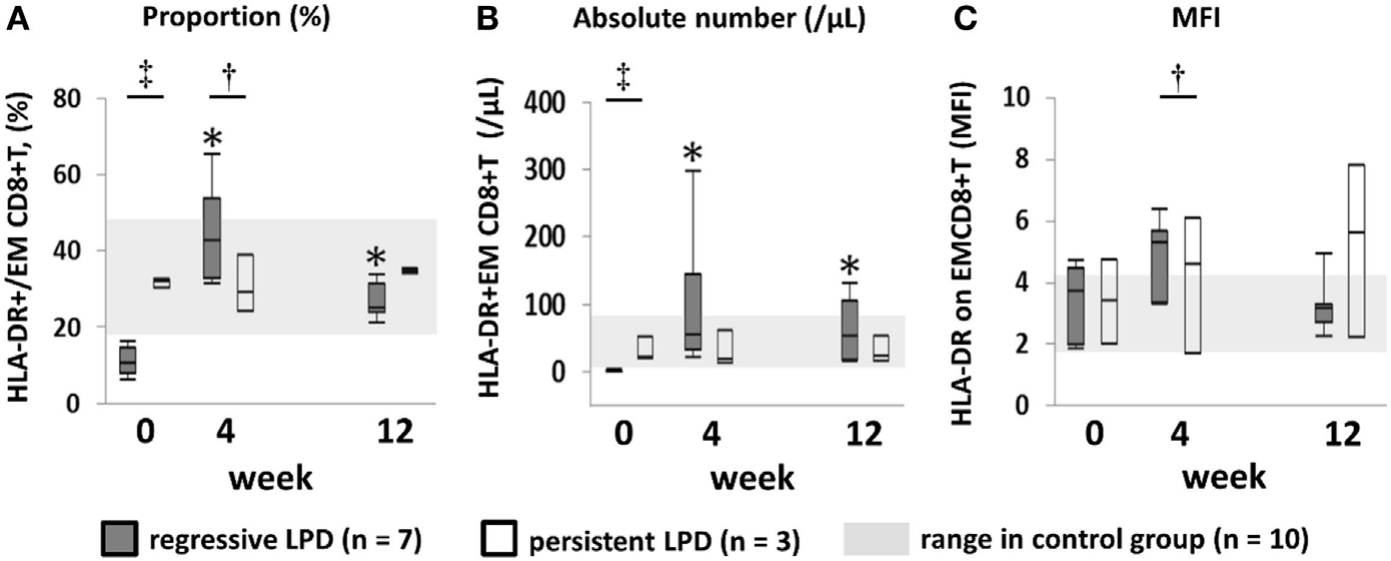
Transition of proportion and absolute number of activated EMCD8+ T cell, mean fluorescence intensity (MFI) of HLA-DR on EMCD8+ T cell after methotrexate (MTX) cessation. Transition of **(A)** proportion and **(B)** absolute number of activated EMCD8+T cell, **(C)** MFI of HLA-DR onEMCD8+ T cell after MTX cessation. Comparison between the three groups was conducted by Kruskal–Wallis test, and comparison between two groups was conducted by Wilcoxon test. ^†^Regressive vs. Control, *P* < 0.05. ^‡^Regressive vs. Control, *P* < 0.05; Regressive vs. Persistent, *P* < 0.05. *P < 0.05 for the comparison with the value at week 0 in each group. EM, effector memory.

Since we expected EMCD8+ T cells to be the key of the anti-LPD effector cells (16), we measured the proportion and absolute numbers of HLA-DR+EMCD8+ T cells to assess the activation status of EMCD8+ T cells. Both the proportion and absolute numbers of HLA-DR+EMCD8+ T cells in the regressive group were significantly lower than those of the persistent and control groups at week 0 (*P* < 0.05 in the comparison between regressive vs. control and regressive vs. persistent), but were significantly increased to higher level than that of control group at week 4, and were at equivalent level with control group at week 12 (Figure 6). On the other hand, while mean fluorescence intensity (MFI) of HLA-DR did not significantly differ among groups at week 0, it significantly increased to a higher level in the regressive group compared with the control group at week 4 (Figure 6C). The proportion and absolute numbers of HLA-DR+EMCD8+ T cells, and MFI of HLA-DR on EMCD8+ T cells, did not show significant changes from weeks 0 to 12 in the persistent group (Figures 6A–C).

### Correlation Between Restoration of Th1 Cells and EMCD8+ T Cells and Change in Serum Cytokine Levels

**Figure 7:**
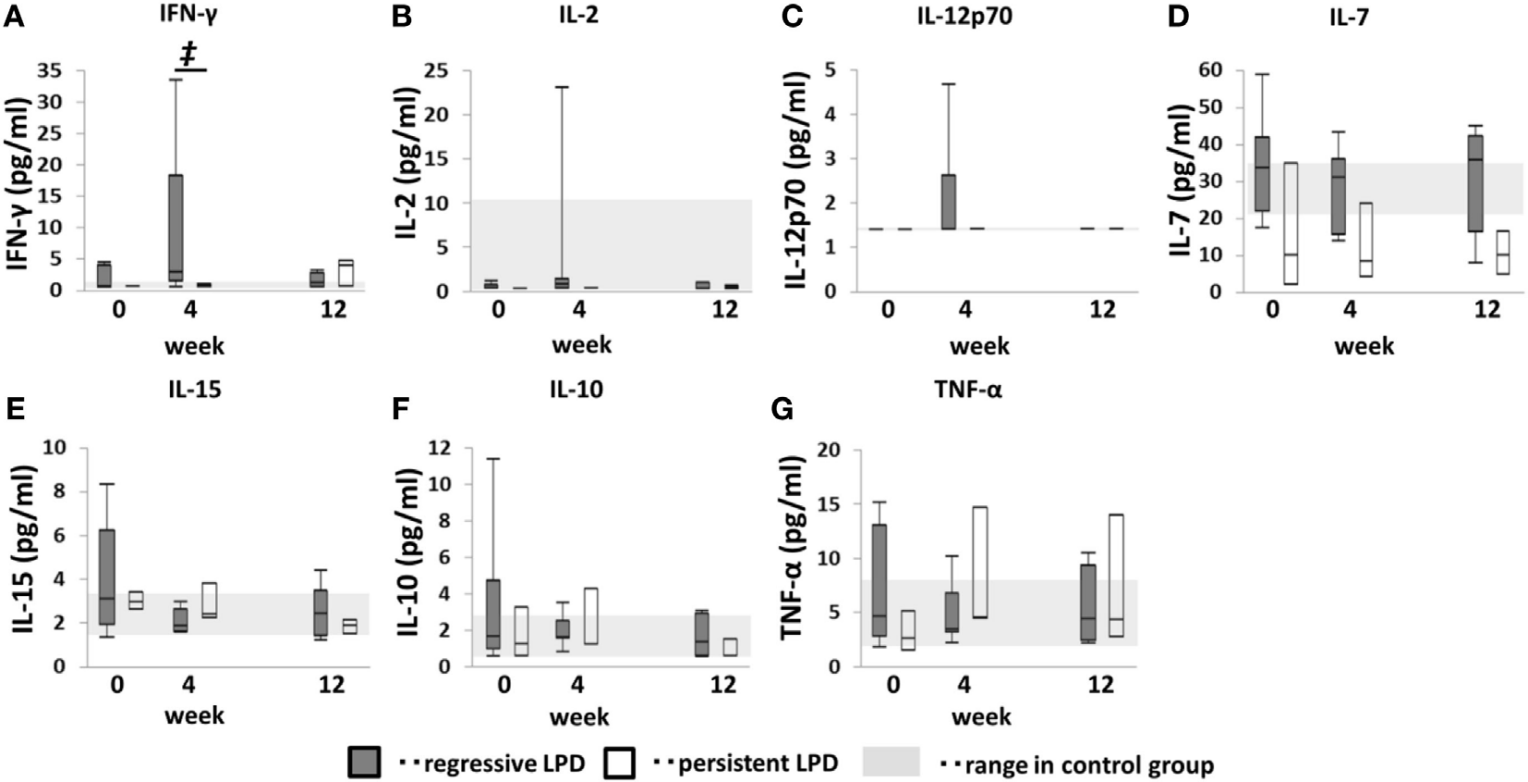
Changes in serum cytokines after methotrexate (MTX) cessation in lymphoproliferative disorder (LPD) patients. Transition of **(A)** interferon (IFN)-γ, **(B)** IL-2, **(C)** IL-12p70, **(D)** IL-7, **(E)** IL-15, **(F)** IL-10, and **(G)** TNF-α after MTX cessation. Comparison between the three groups was conducted using the Kruskal–Wallis test. ^†^Regressive vs. control, *P* < 0.05. ^‡^Regressive vs. control, *P* < 0.05; regressive vs. persistent, *P* < 0.05. *P < 0.05 for comparison with the value at week 0 in each group.

To identify the key cytokine in this process, we measured serum IFN-γ, IL-2, IL-7, IL-10, IL-12p70, IL-15, and TNF-α levels after MTX cessation (Figure 7). Serum IFN-γ levels were not significantly different among groups at week 0, but it increased to significantly higher level than that of persistent and control group at week 4. However, no such increase was observed in the persistent group. IL-2, IL-7, IL-10, IL-12p70, IL-15, and TNF-α levels in both the regressive and persistent groups did not significant change from weeks 0 to 12 and were equivalent to those of the control group. However, IL-15 levels in the regressive group seemed to decrease from weeks 0 to 4 in some cases.

**Figure 8:**
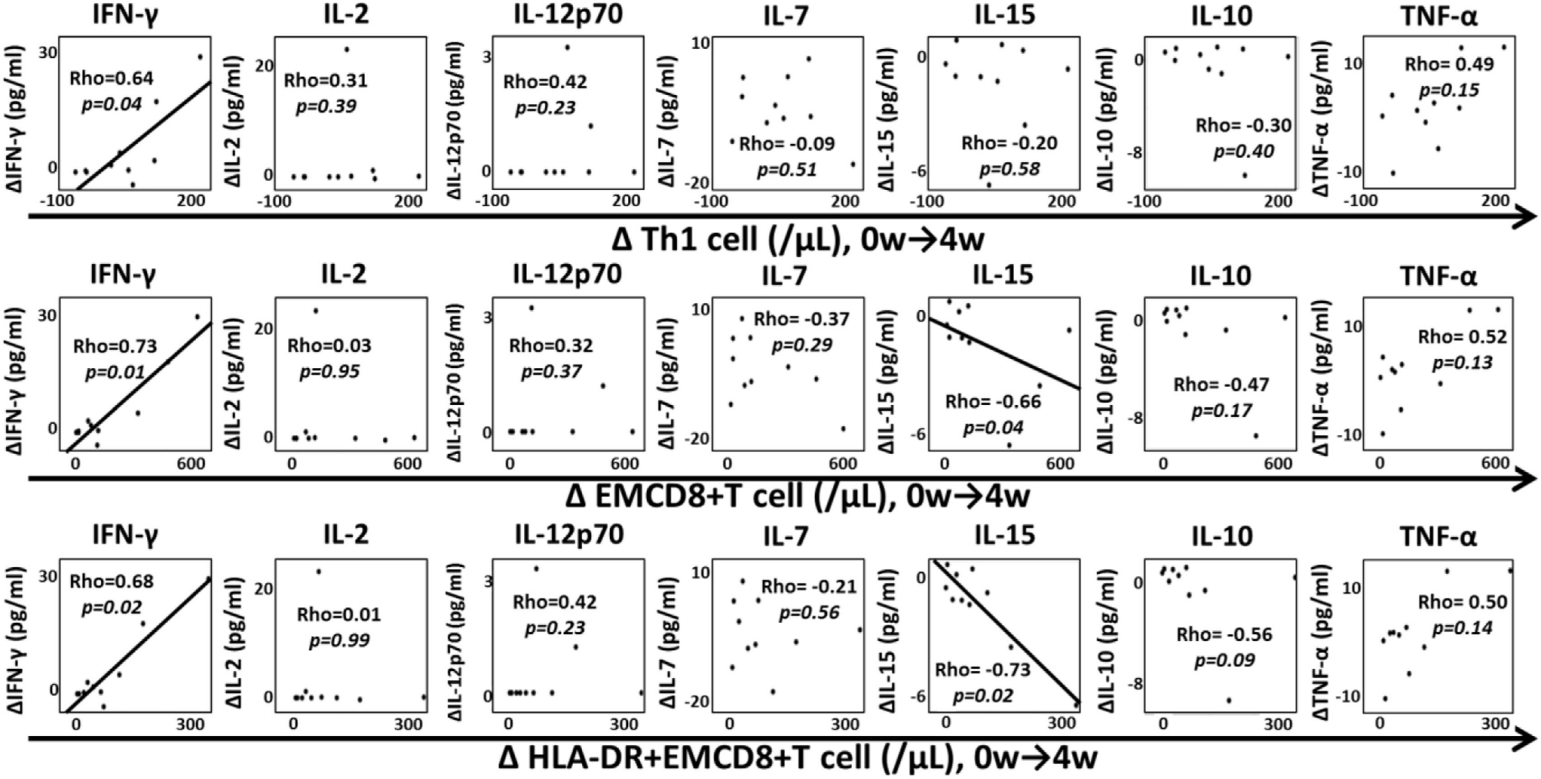
Correlation between increased lymphocyte subsets and cytokine change. Correlation between increase in Th1 cells, EMCD8+ T cells, HLA-DR+EMCD8+ T cells, and changes in interferon (IFN)-γ, IL-2, IL-12p70, IL-7, IL-15, IL-10, and TNF-α from weeks 0 to 4. Correlations were analyzed by Spearman’s correlation coefficient. Th1, T helper 1; EM, effector memory.

We assessed the correlation between changes in Th1 cells, EMCD8+ T cells, HLA-DR+EMCD8+ T cells and each cytokine from weeks 0 to 4 (Figure 8) to investigate the role of cytokines in the expansion of T cell subsets. The increase of IFN-γ (ΔIFN-γ) was significantly correlated with ΔTh1 cells, ΔEMCD8+ T cells and ΔDR+EMCD8+ T cells (*P* < 0.05). In contrast, ΔIL-15 was inversely correlated with ΔEMCD8+ T cells and ΔDR+EMCD8+ T cells (*P* < 0.05). There was no correlation between the other cytokines and the increase in these cell subsets. In addition, there was no significant correlation between ΔIFN-γ, ΔIL-15, and ΔTh2 cells, ΔNK cells, or ΔTh17 cells (data not shown). Transition of quantitative EBV-PCR level after MTX cessation and correlation between change of EBV-PCR and EBV-specific CD8+ T cell were demonstrated in Figure S2 in Supplementary Material.

## Discussion

Previous studies by our group and others (8, 9) have suggested that the decrease in lymphocytes at the time of LPD diagnosis and their restoration following MTX withdrawal may associate with the pathogenesis and regression of LPD developed during MTX administration. Here, we focused on the changes of lymphocyte subsets that were associated with the regression of LPD. Immunophenotyping of peripheral blood cells revealed a restoration of the proportion and absolute numbers of Th1 cells, EMCD8+ T cells and EBV-specific CD8+ T cells during the regression of LPD developed during MTX administration.

Our data also showed an association between the increase in Th1 cells and EMCD8+ T cells and that of IFN-γ after MTX cessation. The lack of such changes in persistent LPD indicates that the pathogenesis of regressive and persistent LPD is discriminable. We also showed that EBV-specific CD8+ T cells decrease at the time of LPD diagnosis and are restored after MTX cessation in regressive EBER-positive LPD patients. A previous study of post-transplant lymphoproliferative disease reported that EBV-specific T cells show an anti-LPD effect even in small numbers (17). Interestingly, the transition of lymphocyte subsets was not significantly different between the pathological phenotypes of LPDs, suggesting a common regression mechanism among each phenotype; however, further studies are needed to confirm this hypothesis.

**Figure 9:**
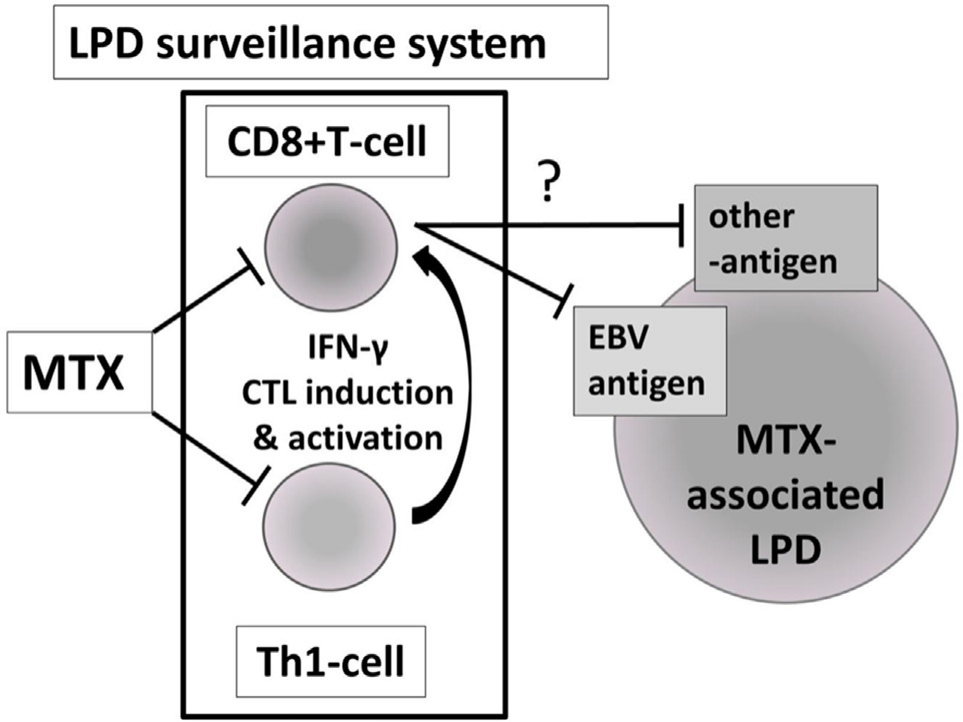
Hypothesis of the pathological and regression mechanism of “regressive LPD” as a narrow sense of “MTX-associated LPD”. MTX, methotrexate; LPD, lymphoproliferative disorder; Th1, T helper 1; EBV, Epstein–Barr virus.

Currently, all LPDs that develop during MTX administration are classified as “other iatrogenic immunodeficiency-associated LPD” according to the latest WHO classification of lymphoid neoplasms (4), regardless of the status of LPD following MTX cessation. This is the first study to report differences in immune status between regressive and persistent LPDs developed during MTX administration in RA patients. Excessive inhibition of Th1 cells, EMCD8+ T cells and EBV-specific CD8+ T cells by MTX at the time of LPD development, and their restoration after MTX cessation appear to be features specific to the pathogenic and regression mechanism of “regressive LPD” (Figure 9), since a decrease in the proportion and absolute number of these cell subsets was not observed in persistent LPD. This suggests that persistent LPD is not caused by inhibition of the “LPD surveillance system”. Therefore, our findings could represent the first evidence of distinct types of MTX-associated LPD: “regressive LPD” and those caused by inhibition of the “LPD surveillance system”.

Th1 cells are one of the major sources of IFN-γ (18); it is assumed that the Th1 response was initiated before the increase of IFN-γ in regressive LPDs. MTX inhibits Th1 response and cytokine production, including IFN-γ (19–21), suggesting that excessive inhibition of Th1 cells by MTX may be involved in the pathogenesis of MTX-associated LPD. Furthermore, the important role of CD4+ T cells in promoting CD8+ T cell proliferation and their response to antigens is well established (22, 23). IL-15 is required for the basal proliferation of memory CD8+ T cells (24); the decrease in IL-15 from baseline after MTX withdrawal, which was correlated with the increase in EMCD8+ T cells, might suggest the IL-15 secretion induced by the decrease of EMCD8+ T cells.

Some limitations of our study warrant mention. First, the number of LPD cases for which peripheral blood was analyzed by flow cytometry was limited because of the low incidence of LPD during MTX administration, even though the number of patients was rather large for such a rare disease. We also could not analyze the difference in cell subsets between each pathological phenotype of LPD because of the small number of patients. Second, as we only focused on the cell subsets that were significantly different in proportion between control and regressive groups, we cannot rule out potential associations between regression of LPD and cell subsets that showed a difference in absolute numbers, such as NK cells, and B cells. Third, we did not examine the anti-LPD function of the cell subsets within the peripheral blood. Previous studies indicate that circulating lymphocyte subsets including cytotoxic CD8+ T cells and EBV-specific CD8+ T cells in a cancer-bearing situation have specific anti-tumor function (16, 25, 26).

In conclusion, assessment of the immunological status of LPD patients treated with MTX enabled to close on the pathogenesis of regressive LPD after MTX cessation. Studies that examine the difference in immunological status between pathological phenotypes of LPDs are warranted in the future.

## Ethics Statement

This study was carried out in accordance with the recommendations of “ethics committee of Keio University School of Medicine”; and the “ethics committee of Saitama Medical Center, Saitama Medical University”; with written informed consent from all subjects. All subjects gave written informed consent in accordance with the Declaration of Helsinki. The protocol was approved by the “ethics committee of Keio University School of Medicine” and the “ethics committee of Saitama Medical Center, Saitama Medical University.”

## Author Contributions

SS performed most of the experiments. SS, KS, MT, and TT participated in the study conception and design of the work. SS, KS, KYo, YK, KYa, TS, TM, SO, KK, KA, JT, MT, and TT participated in the acquisition of data. SS, KS, YK, KYa, MT, and TT participated in the analysis and interpretation of data. SS, KS, Kya, and TT were involved in drafting the manuscript. All authors were involved in revising it critically for important intellectual content and approved the final version to be published. All authors agreed to be accountable for all aspects of the work in ensuring that questions related to the accuracy or integrity of any part of the work are appropriately investigated.

## Conflict of Interest Statement

SS has received speaking fees from Chugai Pharmaceutical, Eisai, and Pfizer Japan. KS has received research grants from Eisai, Bristol-Myers Squibb, Kissei Pharmaceutical, Daiichi-Sankyo, and speaking fees from Abbie Japan, Astellas Pharma, Bristol-Myers Squibb., Chugai Pharmaceutical, Eisai, Fuji Film Limited, Janssen Pharmaceutical, Kissei Pharmaceutical, Mitsubishi Tanabe Pharmaceutical, Pfizer Japan, Shionogi, Takeda Pharmaceutical, UCB Japan, and consulting fees from Abbie, Pfizer Japan. KYo has nothing declared. YK has received research grants or lecture fees from Abbvie, Eisai Pharmaceutical, Chugai Pharmaceutical, Bristol Myers Squibb, Astellas Pharmaceutical, Mitsubishi Tanabe Pharma Corporation, Pfizer, Janssen, and UCB. KYa has received consultant fees from Pfizer, Chugai Pharma, Mitsubishi-Tanabe Pharma, Abbvie; received honoraria from Pfizer, Chugai Pharma, Mitsubishi-Tanabe Pharma, Bristol-Myers Squibb, Takeda Industrial Pharma, GlaxoSmithkline, Nippon Shinyaku, Eli Lilly, Janssen Pharma, Eisai Pharma, Astellas Pharma, Acterlion Pharmaceuticals; and received research support from Chugai Pharma, Mitsubishi-Tanabe Pharma. TS has received speaking fees from Chugai Pharmaceutical Co., Ltd., Eisai, and Janssen. TM has received speaking fees from Astellas Pharma Inc, Chugai Pharmaceutical Co, Ltd., Eisai, Pfizer, and Janssen. SO has received research grants and speaking fees from Astellas Pharma Inc, Chugai Pharmaceutical Co, Ltd., Eisai, Bristol–Myers K.K., Pfizer, Takeda Pharmaceutical Co., Ltd., Teijin Pharma Ltd., and AbbVie GK. KK has nothing to declare. KA has received honoraria from Pfizer Japan and Tanabe-Mitsubishi Pharmaceutical Co. JT has nothing to declare. MT has received speaking fees from Eisai. TT has received research grants from Astellas Pharma Inc, Bristol–Myers K.K., Chugai Pharmaceutical Co, Ltd., Daiichi Sankyo Co., Ltd., Takeda Pharmaceutical Co., Ltd., Teijin Pharma Ltd., AbbVie GK, Asahikasei Pharma Corp., Mitsubishi Tanabe Pharma Co., Pfizer Japan Inc., and Taisho Toyama Pharmaceutical Co., Ltd., Eisai Co., Ltd., AYUMI Pharmaceutical Corporation, speaking fees from AbbVie GK., Bristol–Myers K.K., Chugai Pharmaceutical Co,. Ltd., Mitsubishi Tanabe Pharma Co., Pfizer Japan Inc., and Astellas Pharma Inc, and Daichi Sankyo Co., Ltd, and consultant fees from Astra Zeneca K.K., Eli Lilly Japan K.K., Novartis Pharma K.K., Mitsubishi Tanabe Pharma Co., Abbvie GK, Nipponkayaku Co., Ltd, Janssen Pharmaceutical K.K., Astellas Pharma Inc.

## Supplementary Material

Figure S1 Transition of Th1 cells and EMCD8+ T cells between different pathological classifications of lymphoproliferative disorders (LPDs). Transition of the proportion of Th1 cells among CD4+ T cells in **(A)** regressive LPD and **(B)** persistent LPDs, and transition of the proportion of EMCD8+ T cells among CD8+ T cells in **(C)** regressive LPD and **(D)** persistent LPDs. Th1, T helper 1; EM, effector memory.

Figure S2 Transition of quantitative Epstein–Barr virus (EBV)-PCR level after methotrexate (MTX) cessation and correlation between change of EBV-PCR and EBV-specific CD8+ T cells. **(A)** Transition of quantitative EBV-PCR level after MTX cessation and **(B)** correlation between change of EBV-PCR and EBV-specific CD8+ T cells.
