# Supplementary figures and images for: Restoration of Decreased T Helper 1 and CD8+ T Cell Subsets Is Associated With Regression of Lymphoproliferative Disorders Developed During Methotrexate Treatment
Source: Front Immunol. 2018 Apr 4;9:621. doi: 10.3389/fimmu.2018.00621 (PMC5893782; doi:10.3389/fimmu.2018.00621)

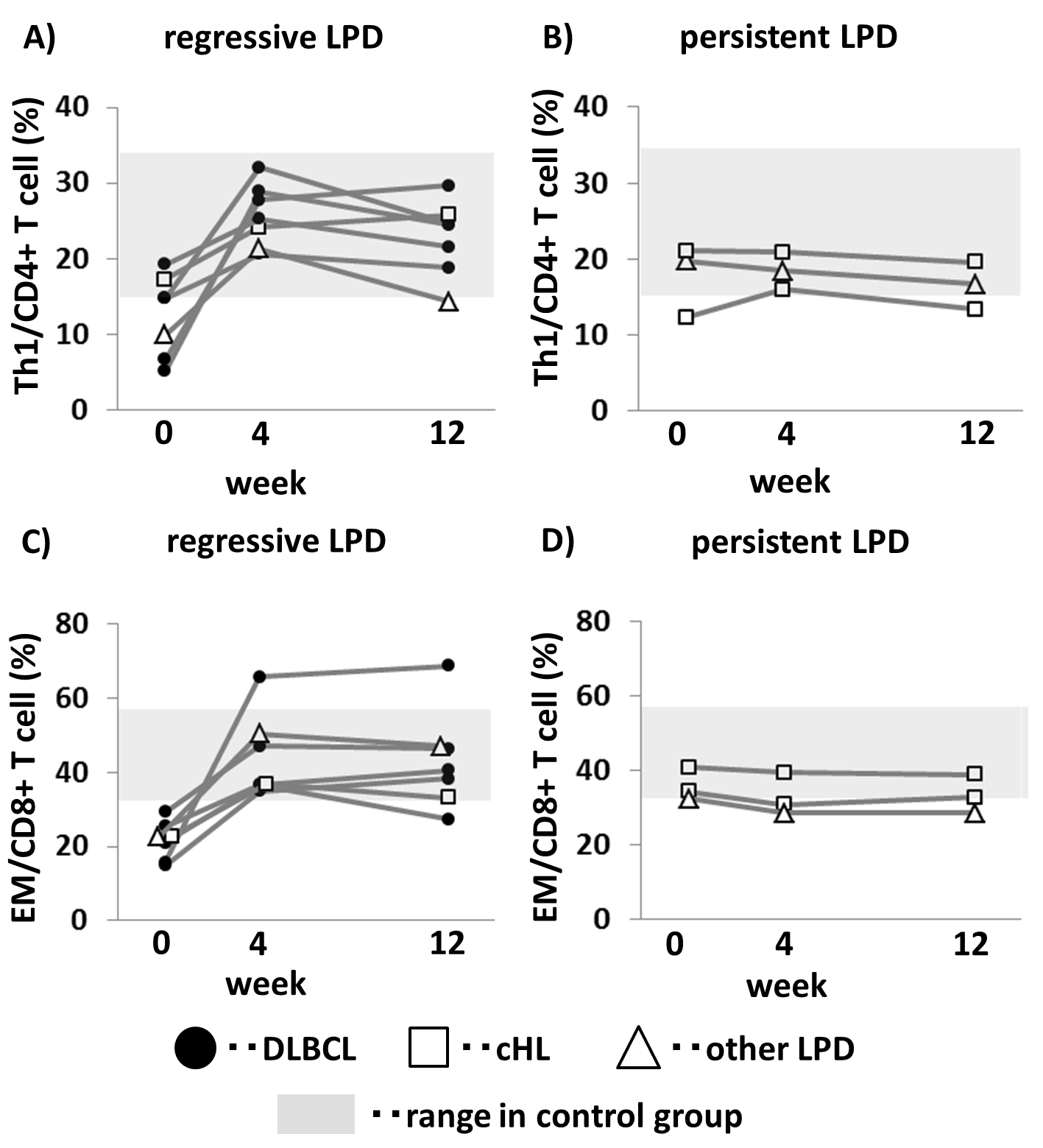

Supplement: Figure S1 — Transition of Th1 cells and EMCD8+ T cells between different pathological classifications of lymphoproliferative disorders (LPDs). Transition of the proportion of Th1 cells among CD4+ T cells in (A) regressive LPD and (B) persistent LPDs, and transition of the proportion of EMCD8+ T cells among CD8+ T cells in (C) regressive LPD and (D) persistent LPDs. Th1, T helper 1; EM, effector memory. [file image_1.tif]

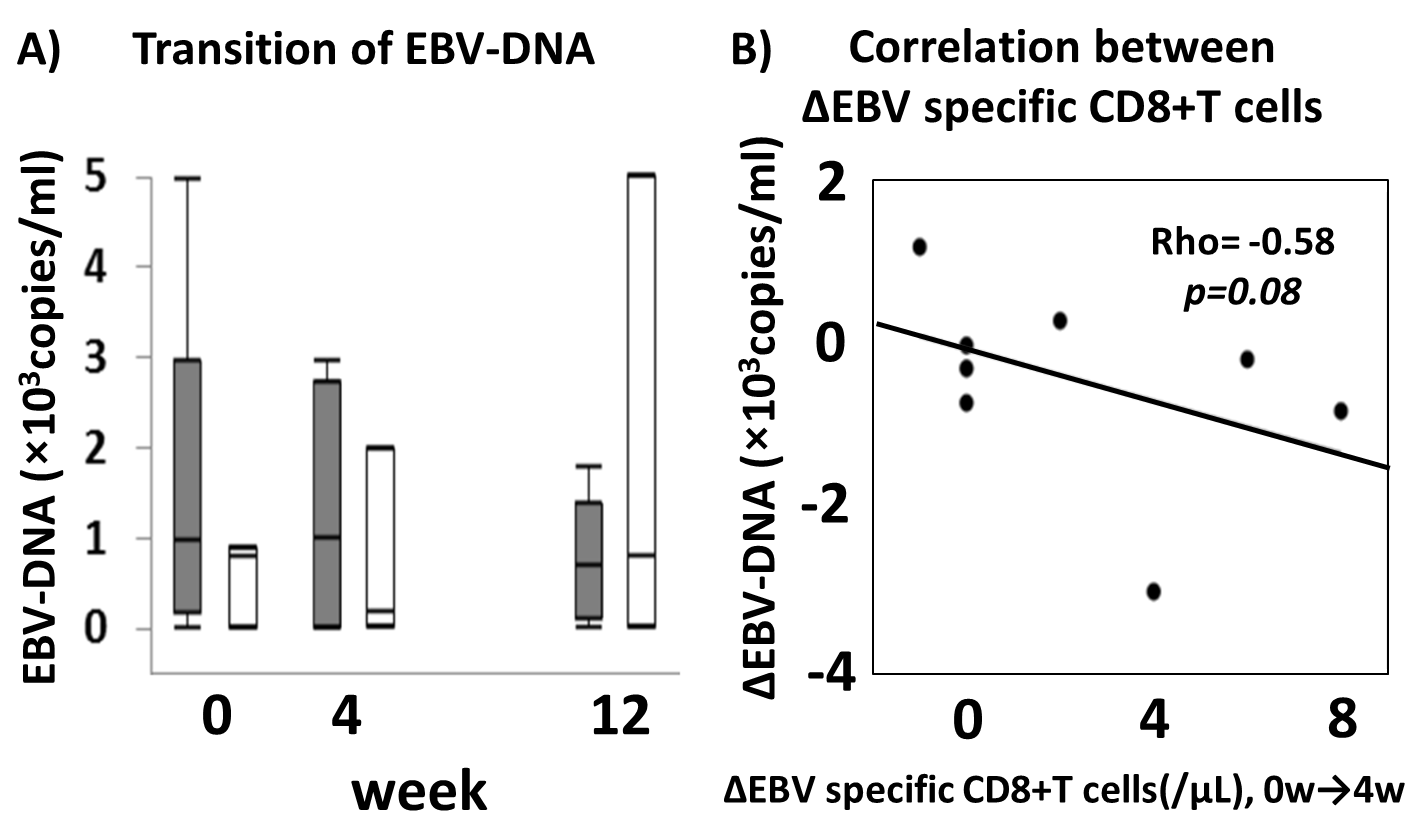

Supplement: Figure S2 — Transition of quantitative Epstein–Barr virus (EBV)-PCR level after methotrexate (MTX) cessation and correlation between change of EBV-PCR and EBV-specific CD8+ T cells. (A) Transition of quantitative EBV-PCR level after MTX cessation and (B) correlation between change of EBV-PCR and EBV-specific CD8+ T cells. [file image_2.tif]
